# Supplementary material for: Exploring the sentiment of entrepreneurs on Twitter
Source: PLoS One. 2021 Jul 30;16(7):e0254337. doi: 10.1371/journal.pone.0254337 (PMC8323876; doi:10.1371/journal.pone.0254337)
Supplement: Attachment — Submitted filename: Response_final.pdf [file pone.0254337.s001.pdf]

<u>Response Letter to Editor and Reviewers [PONE-D-20-15982]</u>

Thank you for submitting your manuscript to PLOS ONE. We have received three excellent reports on your paper and I am very grateful to the highly constructive feedback the referees provided on your paper. As you will see, all three referees see merit in your study but also raise substantial concerns about your analyses, how you interpret your results, and how they fit into the existing literature. All concerns raised by the referees would need to be addressed in a major revision and a point-by-point response before we can consider publication of your work in PLOS ONE. In particular, both R1 and R2 point out that your data and analyses do not allow you to make any claims about how people actually feel or why, partly because of measurement validity issues and partly because of your study design. Furthermore, in response to R1's comments, I'd like to point out that while novelty or a theoretical contribution are not requirements for publication in PLOS ONE, we do require clearly stated research questions and appropriate discussions of your empirical results and how they fit into the existing literature.

Dear Professor Koellinger,

Thank you very much for requesting a revision of our paper and for the helpful comments that you and the reviewers have provided us. We are delighted that all three reviewers see merit in our study. We provide a point-by-point response to the comments of the reviewers below, however, allow us to share here the key changes in the revised version:

1. Following the helpful comments of R1 and R2 we have positioned the paper as a study of the emotional expression of entrepreneurs and non-entrepreneurs on online social media platforms.
2. We have clarified our contributions and our terminology regarding sentiment and emotions.
3. We have expanded the dictionaries for detecting social and serial entrepreneurs, as suggested by R2.
4. Taking into account the comments of R2, we further expanded the details of VADER and uClassify tool that we used for sentiment analysis and topics recognition, respectively.
5. Finally, as R3 suggested we have expanded the prior literature that is related to our study.

We also very much appreciate your clarification that "while novelty or a theoretical contribution are not requirements for publication in PLOS ONE, we do require clearly stated research questions and appropriate discussions of your empirical results and how they fit into the existing literature".

Your comments and those of the reviewers have significantly improved the quality of our paper. Thank you!

Please include the following items when submitting your revised manuscript:

- **A rebuttal letter that responds to each point raised by the academic editor and reviewer(s). You should upload this letter as a separate file labeled 'Response to Reviewers'.**
- **A marked-up copy of your manuscript that highlights changes made to the original version. You should upload this as a separate file labeled 'Revised Manuscript with Track Changes'.**
- **An unmarked version of your revised paper without tracked changes. You should upload this as a separate file labeled 'Manuscript'.**

If you would like to make changes to your financial disclosure, please include your updated statement in your cover letter. Guidelines for resubmitting your figure files are available below the reviewer comments at the end of this letter.

If applicable, we recommend that you deposit your laboratory protocols in protocols.io to enhance the reproducibility of your results. Protocols.io assigns your protocol its own identifier (DOI) so that it can be cited independently in the future. For instructions see: http://journals.plos.org/plosone/s/submission-guidelines#loc-laboratory-protocols

We look forward to receiving your revised manuscript.

Kind regards,
Philipp D. Koellinger, Ph.D.
Academic Editor
PLOS ONE

Journal requirements:

When submitting your revision, we need you to address these additional requirements.

1. Please ensure that your manuscript meets PLOS ONE's style requirements, including those for file naming. The PLOS ONE style templates can be found at https://journals.plos.org/plosone/s/file?id=wjVg/PLOSOne_formatting_sample_main_body.pdfa nd https://journals.plos.org/plosone/s/file?id=ba62/PLOSOne_formatting_sample_title_authors_affiliations.pdf

2.We note that you have indicated that data from this study are available upon request. PLOS only allows data to be available upon request if there are legal or ethical restrictions on sharing data publicly. For more information on unacceptable data access restrictions, please see http://journals.plos.org/plosone/s/data-availability#loc-unacceptable-data-access-restrictions.

In your revised cover letter, please address the following prompts:

a) If there are ethical or legal restrictions on sharing a de-identified data set, please explain them in detail (e.g., data contain potentially sensitive information, data are owned by a third-party

organization, etc.) and who has imposed them (e.g., an ethics committee). Please also provide contact information for a data access committee, ethics committee, or other institutional body to which data requests may be sent.

b) If there are no restrictions, please upload the minimal anonymized data set necessary to replicate your study findings as either Supporting Information files or to a stable, public repository and provide us with the relevant URLs, DOIs, or accession numbers. For a list of acceptable repositories, please see http://journals.plos.org/plosone/s/data-availability#loc-recommended-repositories.

We will update your Data Availability statement on your behalf to reflect the information you provide.

3. We note you have included a table to which you do not refer in the text of your manuscript. Please ensure that you refer to Table 10 in your text; if accepted, production will need this reference to link the reader to the Table.

Comments to the Author

1. Is the manuscript technically sound, and do the data support the conclusions?

The manuscript must describe a technically sound piece of scientific research with data that supports the conclusions. Experiments must have been conducted rigorously, with appropriate controls, replication, and sample sizes. The conclusions must be drawn appropriately based on the data presented.
Reviewer #1: Partly
Reviewer #2: Partly
Reviewer #3: Yes

---

2. Has the statistical analysis been performed appropriately and rigorously?
Reviewer #1: Yes
Reviewer #2: Yes
Reviewer #3: Yes

---

3. Have the authors made all data underlying the findings in their manuscript fully available?

The PLOS Data policy requires authors to make all data underlying the findings described in their manuscript fully available without restriction, with rare exception (please refer to the Data Availability Statement in the manuscript PDF file). The data should be provided as part of the manuscript or its supporting information, or deposited to a public repository. For example, in addition to summary statistics, the data points behind means, medians and variance measures should be available. If there are restrictions on publicly sharing data—e.g. participant privacy or use of data from a third party—those must be specified.
Reviewer #1: No

Reviewer #2: No
Reviewer #3: No

---

4. Is the manuscript presented in an intelligible fashion and written in standard English?

PLOS ONE does not copyedit accepted manuscripts, so the language in submitted articles must be clear, correct, and unambiguous. Any typographical or grammatical errors should be corrected at revision, so please note any specific errors here.
Reviewer #1: Yes
Reviewer #2: Yes
Reviewer #3: Yes

---

5. Review Comments to the Author

Please use the space provided to explain your answers to the questions above. You may also include additional comments for the author, including concerns about dual publication, research ethics, or publication ethics. (Please upload your review as an attachment if it exceeds 20,000 characters)

Reviewer #1:

This is a well written paper that explores entrepreneurs' emotions based on an innovative big data set from Twitter. I very much appreciate the authors' efforts to analyze Twitter data because Twitter data can be a very valuable data source. I also like the approach of matching the Twitter data with data from Crunchbase. Nevertheless, I have some concerns about the paper in its current form:

Thank you very much for your helpful and constructive comments. We are very happy that you found the paper to be well written and that you liked our matching of Twitter and Crunchbase data. Your comments have significantly improved the quality of our paper. Please find below your comments in bold and our responses in plain text.

1. My first major concern is that I don't understand what it is you bring new to the table. In more formal words, what is (are) the gap(s) that you address with your study? At the beginning of the introduction, you motivate well the phenomenon under study and provide a detailed and extensive overview of the current knowledge of the topic. However, I am missing clear statements about what we still don't know about entrepreneurs' emotions, why it is important to study these gaps, and how exactly you address these gaps with your study. For me, it is absolutely essential that you state clear answers to these three points before you explain the details of your study. Therefore, I do not see any contributions of your study to the field at the moment. This assessment is further corroborated when reading your research questions. In your review of the current literature in the introduction, you can already answer those questions. Why do you need to study these questions again? To answer this question, I suggest you think mainly about theoretical reasons. It appears that you argue that your contribution is to answer these questions with

an innovative data set, but this contribution would also need to be supported with a theoretical gap.

Thank you for this important comment, which gave us the opportunity to further clarify our contributions. Please see below and pages 5-6:

"Our work complements earlier studies using Twitter data in managerial or entrepreneurial studies [17,34–36] and makes a number of contributions: Our first contribution is to examine whether there are differences between categories of entrepreneurs (traditional, social and serial entrepreneurs) in their emotional expression on social media platforms like Twitter. Most of the previous research focused on entrepreneurs' emotions but not on the emotional expression of entrepreneurs in social media platforms. In addition, our study expands previous work that considers how emotion influences social entrepreneurship intention and participation [40–46] and shows that social entrepreneurship can lead the entrepreneur to express more positive emotions than other forms of entrepreneurship on social media platforms. We also contribute to the limited literature on emotions in serial entrepreneurship [47,48] by showing that serial entrepreneurship can lead to the expression of less positive emotions than other forms of entrepreneurship. Our second contribution is to compare differences in emotions expressed on Twitter between entrepreneurs and non-entrepreneurs in relation to different topics. There is currently limited work examining the effect of entrepreneurship on non-entrepreneurial emotions. We build on prior literature that has considered emotions specific to certain topics, such as Cardon et al.'s [22] and Gielnik et al.'s [49] examination of emotions towards entrepreneurial identity and activity. By employing 10 different topics, namely "Society", "Recreation", "Health", "Business", "Home", "Science", "Computers", "Games", "Arts", "Sports, we show that entrepreneurs express significantly more positive emotions than non-entrepreneurs in all topics. Our third contribution is to examine how transition from entrepreneurship to non-entrepreneurship and vice versa can affect the emotions of an individual on social media platforms responding to recent calls [50,51]; we show that engagement in entrepreneurship is associated with a higher emotional positivity. These findings further support the argument that entrepreneurship is associated with more positive emotional expressions on social media platforms. Finally, our study contributes to recent research that has examined the digital footprints of entrepreneurs using computerized text analyses [47][48][49]."

2. I am not sure I agree with some of your main statements in the paper. First, I am not sure that you actually study entrepreneurs' emotions, but that you rather study their emotional expression on a social media platform. These expressions are likely to be influence by social desirability and you somehow need to account for this or at least be more careful and precise with your statements (see for example the book by Seth Stephens-Davidowski, 2017). In addition, expressing positive emotions on Twitter can also be a form of cognitive dissonance, i.e., entrepreneurs express positive emotions to justify their choice to become (social, serial) entrepreneurs. Second, you overstate your findings in (implicitly) drawing generalizations about all entrepreneurs when in fact you analyze entrepreneurs from London and Los Angeles. You actually analyze data from entrepreneurs in the biggest entrepreneurship ecosystems, hence you can only draw generalizations about such entrepreneurs (please see Yarkoni, 2020, about the generalizability crisis). Referring to my previous point, it may also be likely that especially these entrepreneurs express positive

emotions because they are supposed to express positive emotions as entrepreneurs in one of these start-up hubs.

Thank you for this very helpful comment.

In the revised paper, we have positioned the study as suggested and we now state clearly that we study the emotional expressions of entrepreneurs on online social media platforms.

Please see Introduction and pages 4-6:

"...We examine differences between entrepreneurs and non-entrepreneurs in their emotional expression on social media platforms. We examine differences in emotions across novice, social and serial entrepreneurs. Finally, we examine how transition into entrepreneurship can influence an individual's emotions on social media platforms."

"...Our first contribution is to examine whether there are differences between types of entrepreneurs in their emotional positivity on social media platforms by looking at the emotions of social and serial entrepreneurs."

"...We argue that social entrepreneurship can lead the entrepreneur to experience more positive emotions on social media platforms than other forms of entrepreneurship, complementing and extending previous work that considers how emotion influences social entrepreneurship intention and participation [36–42]."

"...We also contribute to the limited literature on emotions in serial entrepreneurship [43,44] by showing that serial entrepreneurship can lead to less positive emotions on social media platforms than other forms of entrepreneurship."

"...Our second contribution is to compare differences in emotions expressed on Twitter between entrepreneurs and non-entrepreneurs in relation to different topics."

"...Our third contribution is to examine how transition from entrepreneurship to non-entrepreneurship and vice versa can affect the emotions of an individual on social media platforms responding to recent calls [46,47]."

Please see Theory and Research Questions and pages 6-10:

"This subsection compares the emotional expressions of entrepreneurs and non-entrepreneurs on social media platforms...In summary, the Research Questions (RQs) are the following ones: RQ1: Are entrepreneurs more likely than non-entrepreneurs to exhibit positive emotions on social media platforms? RQ2: How does a job change from entrepreneur to non-entrepreneur and vice versa affect the emotions of an individual on social media platforms?"

"...Social entrepreneurs apply business practices to address social problems or create social value, although there are numerous variations on this definition [57–60]. We build on literature that has examined the motivations [41,42] and compassion [37] of social entrepreneurs to examine how their emotions on social media platforms compare to traditional and serial entrepreneurs...In summary, we investigate the following Research Question (RQ): RQ3: Are social entrepreneurs more likely than other entrepreneurs to exhibit positive emotions on social media platforms?"

"This subsection considers the emotions on social media platforms of serial entrepreneurs - entrepreneurs who establish multiple businesses over time [65,66]...In summary, we investigate

the following Research Question (RQ): RQ4: Are serial entrepreneurs less likely than other entrepreneurs to exhibit positive emotions on social media platforms?"

Please see Results and page 21:

“The regression results are shown in Table 5. The dependent variable is the average sentiment of a user’s tweets. Columns 1, 2, and 3 show the results for the London, Los Angeles, and worldwide samples, respectively. The coefficient of "Entrepreneur" is positive and significant in all samples ($p<0.001$), indicating that entrepreneurs express more positive emotions on social media platforms than non-entrepreneurs, providing support for the first research question (RQ1).”

Please see Discussion and pages 29-31:

"Our paper examined whether entrepreneurs are more likely than non-entrepreneurs to express positive emotions on social media platforms. We also examined whether social entrepreneurs are more likely than other entrepreneurs to express positive emotions on social media platforms and whether serial entrepreneurs are less likely to express positive emotions on social media platforms. Using a two-study design with four samples we found that entrepreneurs express more positive emotions on social media platforms relative to non-entrepreneurs. We further showed that social entrepreneurs express more positive emotions relative to other entrepreneurs. We also find that a job change from entrepreneur to non-entrepreneur and vice versa affects the emotions of an individual on social media platforms."

"...We show that social entrepreneurship can lead to more positive emotions on social media platforms than other forms of entrepreneurship, extending prior research arguing that emotion can influence social entrepreneurial intention and participation [36,37,40]."

"Secondly, we show that transition from entrepreneurship to non-entrepreneurship and vice versa affects the emotional expression on social media platforms of an individual, supporting earlier empirical findings by Baron et al. [16] and Tata et al. [17]..."

"Thirdly, we compare differences in emotions on social media platforms between entrepreneurs and non-entrepreneurs in relation to different topics..."

Please see Conclusion and pages 33-34:

“To sum up, our paper has examined differences in the emotional expression on social media platforms of traditional, social, and serial entrepreneurs using much larger samples than have been used in previous studies of entrepreneurial emotion...”

Regarding your second point, in study 1 we examined whether entrepreneurs express more positive emotions than non-entrepreneurs using 3 samples: 1) London, 2) Los Angeles and 3) Worldwide. Furthermore, in study 2, we used individuals from Crunchbase, that are located worldwide. We found that that transition from non-entrepreneurship to entrepreneurship and vice versa affects the emotions expressed by an individual, with a transition to entrepreneurship associated with the expression of more positive emotions. We also mentioned the following limitation in the discussion (page 33):

"…As two of our samples were in the entrepreneurial ecosystems of London and Los Angeles, our results cannot be generalized outside these ecosystems [126]."

3. It appears that you use emotions, positive emotions, and sentiment as substitutes. While sentiment is in the paper's title, the literature review is about positive emotions, and the measure is about emotion and sentiment as substitutes. From your description of the variable "emotion", I am not clear what exactly you measure and which levels your dependent variable has. Please refine the description of your dependent variable and align your theoretical arguments with the operationalization. Please also note that current emotion literature argues that positive and negative emotions are no opposites, but that they are orthogonal. People can experience positive and negative emotions at the same time.

Thank you for this important comment, which contributed to clarify our terminology.

Please see below and page 4:

"In this work, we examine differences in the emotional expression of entrepreneurs versus non-entrepreneurs as manifested in their messages ("tweets") on Twitter, the major online platform for public expression. We apply emotion mining, a subtask of sentiment analysis [37–39], to determine the polarity of emotions in tweets. Sentiment and emotion are closely related concepts of Natural Language Processing with sentiment reflecting the emotion that 'colors' an expressed opinion or idea. Sentiment analysis techniques deduce a writer's emotions and opinions through Natural Language Processing. Applied on Twitter, sentiment analysis algorithms take the posted text, emoticons and hashtags of each tweet and return a score ranging from -1 (extreme negative emotions) to 1 (extreme positive emotions). Text is the main medium of Internet-mediated human communication and, therefore, analysing the sentiment and the topic of tweets at a massive scale can provide important observations regarding the emotions that groups of users express on Twitter, either in general or about specific topics. A taxonomy of sentiment analysis and a complete survey on emotion theories is presented in Yadollahi et al. (2017) [33]."

We also mention the following on page 15:

"In this study, we focus on two emotional states of a tweet: positive and negative. Specifically, as a dependent variable we use the VADER's score that indicates the negativity or positivity of a tweet. VADER incorporates word-order sensitive relationships between terms and is able to determine the magnitude of intensity through punctuation, capitalization, degree modifiers, negations, slang etc. The output of VADER are the positive, negative, and neutral ratios of sentiment. VADER's score ranges from -1 (extreme negativity) to 1 (extreme positivity).

VADER score: $-1 \leq$ Negative sentiment (e.g. -0.3) < 0 (Neutral Sentiment) $<$ Positive sentiment (e.g. 0.2) ≤ 1

Furthermore, in cases where a tweet contains positive and negative emotions at the same time, VADER sums up the strength/intensity of the sentiment for each word in the text and finds the more dominant emotion. For example, if a sentence contains equal intensities scores of positive and negative emotions, then the output of VADER is 0. Overall, VADER's score

determines the emotion intensity in a continuous scale based on the emotional changes in a sentence, and not just the binary polarity (i.e., either positive or negative)."

4. I wonder why you analyze London and Los Angeles separately. If they are – as you argue – similar clusters, why not analyze them together?

Thank you for this excellent comment. In the revised paper, we first clarify the reasons that we selected the three samples. We have added the following in the Study 1 section (pages 12-13):

"We examined London and Los Angeles as they are among the most successful regions in the world in attracting start-ups and venture investors with high rates of entrepreneurial growth and success in attracting funding. Both geographic areas are leading centres of innovation and entrepreneurial hubs[1][2][3][4] [87] in Europe and the United States, respectively. Furthermore, we selected the specific regions as users from these geographic areas are fluent in English and thus the misspellings in the tweets could be less."

Second, we have added the following column in table 5 in the results section (page 21, table 5, column 3), that shows that the results remain the same even when we include both LO and LA in the same regression:

Table A.

**Sample**<br><br>**Region**	**All**<br>**London and Los Angeles**<br>**(3)**
Entrepreneur	.047109***
	(.001899)
Social Entrepreneur	
Serial Entrepreneur	
Number of followers (000s)	.000113***
	(.000029)
Number of followings (000s)	.000269***
	(.000075)
Total number of tweets (000s)	-.000523***
	(.000052)
Android source	-.025788***
	(.003846)
Retweets	-.031764***
	(.005052)
Geotagged tweets	-.052945***

[1] https://www.inc.com/minda-zetlin/best-cities-for-entrepreneurs-austin-dallas-la-san-diego-miami.html

[2] https://www.crowdspring.com/blog/startups-entrepreneurs-best-startup-cities-us/#losangeles

[3] https://londonlovesbusiness.com/london-is-still-top-for-entrepreneurship/#:~:text=London%20continues%20to%20be%20the,the%20UK's%20high%2Dgrowth%20companies.&text=However%2C%20growth%20levels%20among%20young%20enterprises%20are%20down.

[4] https://www.wired.com/insights/2014/12/london-innovation

	(.006564)
Hashtags	.041932***
	(.002047)
Constant	.135881***
	(.002050)
N	11,410
R-squared	0.137
F	226.83***

Notes. The dependent variable is average sentiment based on the Twitter user's tweets. Standard errors are in parentheses.
$^{*}p < .05$; $^{**}p < .01$; $^{***}p < .001$.

5. The structure of the discussion can be improved in that the contributions are not only repeated in a longer format from the introduction, but that you explain specifically how your results close gap(s) in current knowledge and advance the field.

We have significantly revised the discussion and contribution sections - please see pages 29-34.

I wish you all the best with your research and I hope my comments have been helpful.

Thank you very much for your most helpful comments and for sharing your expertise!

Reviewer #2:

The authors present two studies in which they investigate how types of entrepreneurs differ in their emotional expressions on Twitter. In the first study Twitter data is analyzed using VADER and uClassify. The second study combines the Twitter data with background information on the entrepreneurs to be able to investigate the endogeneity (positive people could be more likely to become entrepreneurs). I like how they make use of preprogrammed NLP functions with careful selection of meaningful data samples, count characteristics of the data and how this is integrated in their regression analysis. However, I do have some points that I would like to be clarified and improved.

Thank you very much for your helpful and constructive comments! They have significantly improved the quality of our paper. Please find below your comments in bold and our responses in plain text.

1. People active on twitter are not representative of wider population. How many unsuccessful entrepreneurs are active on twitter? I can imagine that there is a strong self-selection of successful entrepreneurs. People are unlikely to be negative about a choice they have made (such as failing an entrepreneurship). Also, it seems that non-entrepreneurs are less active on twitter than entrepreneurs, which might reflect how twitter is used to promote their own business. This might drive the results.

Thank you for this important comment. We addressed it in the following ways.

First, we mention that we examine differences between entrepreneurs and non-entrepreneurs in their emotional expression on *social media platforms*.

Second, to avoid introducing bias in our results, we control for several factors related to popularity and reputation on Twitter that may affect the sentiment of users. Specifically, we include the following 7 control variables from Twitter: Number of followers, Number of followings, Total number of tweets, Android source tweets, Retweets, Geotagged tweets and Hashtags (please see pages 17-18).

Third, to test whether there is a strong self-selection of successful entrepreneurs on Twitter, we utilized data from Crunchbase and combined them with the Twitter sample from our first study. Crunchbase is a leading online database collecting extensive information on the start-up ecosystem. Crunchbase is maintained by TechCrunch and works with a plethora of partners (venture capital firms and AngelList) to ensure that its data is accurately represented.

Based on the entrepreneurs in our WW (worldwide) Twitter sample and their Twitter username, we identified the name of their company through Crunchbase. We then examined whether an entrepreneur received funding for his company or not. We ended up with 4,648 entrepreneurs that did not received funding (non-successful entrepreneurs) and 1,253 entrepreneurs that received funding (successful entrepreneurs).

We ran a t-test to compare the Twitter activity between entrepreneurs that received funding and those who did not. The results show that there is not a significant difference between successful and unsuccessful entrepreneurs in terms of the number of followers and followings.

Twitter Activity Variables	Unsuccessful Entrepreneurs (have Not received funding)		Successful Entrepreneurs (have received funding)		t	Sig. (2-tailed)
	Mean	SD	Mean	SD		
Number of followers	8518.636	1147.273	9309.571	2168.573	-0.3189	0.7498
Number of followings	1307.785	63.4123	1217.835	107.0901	0.6703	0.5027

2. You compare social with serial entrepreneurs, but also discuss traditional entrepreneurs. Why did you not include them in your analysis? Is it possible that these are the seasoned, successful entrepreneurs, as they are longer holding up the same business? This might make them score higher than the others in terms of a) success and b) pride. Both of them result in positive emotions. I also think the classification of social and serial entrepreneurs could be more sophisticated, namely using a dictionary that reflects the meaning of 'social' and 'serial'.

Thank you for this very important comment. It shows that we were not clear at all in the previous version of the paper and have revised the paper accordingly. In study 1 of our paper, we examine the differences in emotional expressions on social media platforms between traditional, social and serial entrepreneurs. We first compare the emotional expressions of traditional entrepreneurs and non-entrepreneurs and find that entrepreneurs express more positive emotions than non-entrepreneurs (please see pages 20-21). Next, we compare traditional entrepreneurs with social and serial entrepreneurs (please see pages 21, 24 and 25). Our results show that social entrepreneurs express significantly more positive emotions on social media platforms than traditional entrepreneurs, while serial entrepreneurs express more negative emotions than traditional entrepreneurs although the latter result is not significant.

In addition, we have expanded the dictionary that we used for detecting social and serial entrepreneurs and rerun our analysis for study 1. Overall, our results remain the same.

Please see Table below and page 26:

"We also re-run the regressions using a broader description of serial and social entrepreneurs. Specifically, we re-identified social entrepreneurs as users who have in their personal Twitter description the keyword "entrepreneur" and any of the following terms: "social", "social good", "social venture", "non-profit", "philanthropist" or "philanthropy" (N=1732). We re-identified serial entrepreneurs as users who have in their personal Twitter description the keyword "entrepreneur" and any of the following terms: "serial" or "experienced entrepreneur" (N=507). The results were qualitatively similar." Please see the Table below.

Sample	Entrepreneurs only Worldwide
Entrepreneur	
Social Entrepreneur	.008024*** (.001619)
Serial Entrepreneur	-.004093 (.002896)
Number of followers (000s)	2.99e-06 (8.10e-06)
Number of followings (000s)	.000721*** (.000081)
Total number of tweets (000s)	-.000275*** (.000035)
Android source	-.025456*** (.003806)
Retweets	.002393 (.002831)
Geotagged tweets	-.064748*** (.006462)
Hashtags	.010647*** (.001291)
Constant	.144660*** (.000694)
N	24,573
R-squared	0.0150
F	41.63***

Notes. The dependent variable is average sentiment based on the Twitter user's tweets. Standard errors are in parentheses.
$^{*}p < .05$; $^{**}p < .01$; $^{***}p < .001$.

3. Autonomy and engagement with the tasks are offered as an explanation of the effect (mid page 5), but did you try to find this in the tweets? I suspect that a relatively simple dictionary approach could give some important insights of why you see that some entrepreneurs express more positive emotions than others.

Thank you for this comment. We have included this in the directions for future research (please see page 32).

The engagement of entrepreneurs with their tasks is also shown from the topic analysis that we conducted in tables 7, 8 and 9. The three tables present the topics comparison between entrepreneurs and non-entrepreneurs for the 3 datasets (London, Los Angeles and worldwide)

and show that entrepreneurs post around 3-4 times more tweets related to business related matters than non-entrepreneurs.

4. As all entrepreneurs are likely to experience autonomy and engagement, how is the warm glow different for social entrepreneurs? I can imagine that the warm glow also appears in entrepreneurs that feel engaged with less social causes. The way how it is posed now is subjective; social entrepreneurs can fulfill social norms, but other entrepreneurs cannot as they do not pursue social causes. However, some social groups appreciate money, leading to social norms of earning a lot of money as opposed to having a social goal in mind. To avoid this subjectivity, I think I would replace 'warm glow' with gratitude which you receive when you do something that others did not ask for, and that is appreciated. This is probably more often the case with social entrepreneurs than other entrepreneurs.

Thank you for your suggestion. We have replaced "warm-glow" with gratitude as suggested. Please see below and pages 8-10:

"Social entrepreneurs apply business practices to address social problems or create social value, although there are numerous variations on this definition [61–64]. We build on literature that has examined the motivations [45,46] and compassion [41] of social entrepreneurs to examine how their emotions expressed on social media platforms compare to traditional and serial entrepreneurs. As both traditional entrepreneurs and social entrepreneurs have substantial freedom to set their own goals and working conditions, the arguments that we presented on why traditional entrepreneurs show more positive emotions continue to apply to social entrepreneurs. However, there are additional reasons why social entrepreneurs' emotions may be even more positive than those of traditional entrepreneurs. We propose that social entrepreneurship may lead to altruistic enjoyment of other people's improved circumstances, and so raises the social entrepreneur's emotions. We then argue that social entrepreneurs may receive gratitude from others as they are willing to take on the risk and effort to create positive changes in society through their initiatives, and we see how social entrepreneurship may raise social esteem and enable people to meet social norms, leading to more positive emotion.

Social entrepreneurs aim to create social value and will usually derive pleasure from seeing their goals met. This is an example of altruistic enjoyment, where someone derives pleasure from improvements in another person's situation [65]. Altruistic enjoyment has been associated with activation of reward centres in the brain [66]. Altruistic social entrepreneurs would feel pleasure, which is likely to influence their expressed emotions.

Also, a social entrepreneur may experience pleasure from rises in their social esteem or by fulfilling social norms through their activities. A social entrepreneur works to create social value in some way and can be considered to forego personal income or leisure in order to devote their time to the activity. As their work will typically be visible, it can signal to others their generosity and respect for social duty. Many people will try to demonstrate that they are meeting their social duty or fulfilling norms of behaviour [67,68], and the demonstration can bring increased public esteem or protect the social entrepreneur from sanction for neglecting their societal duties. The social entrepreneur's actions may also allow them to meet their personal norms of good behaviour and bring them satisfaction or allow them to avoid guilt."

5. VADER: although it is successful, it is unable to handle negations, sarcasm, misspellings and certain jargon. How do you deal with this? Did you try POS tagging to dig deeper into the data?

Thank you for this very important comment. In the revised paper, we have included all the preprocessing steps that we followed. Please see below and pages 14-15:

"Before utilizing VADER tool for finding the sentiment of each tweet, we followed several preprocessing steps. Specifically, we removed urls, numbers (i.e. numerical text), common symbols (e.g. "=", except the main punctuations e.g. ?!;.,'"), jargon symbols or text (e.g. "&"). Afterwards, we replaced 3 (or more) duplicate characters (e.g. "tooool" => "tool"). Then, we replaced the contractions with the original words (e.g. " I'm " -> " I am ")."

In terms of handling negations, as stated in the paper of Gilbert & Hutto (2014) and their Github repository (https://github.com/cjhutto/vaderSentiment), VADER can handle typical use cases of negation (e.g. "not good", "wasn't very good", etc.). Thus, we have clarified this below and pages 14-15:

"VADER can handle typical use cases of negation like "not good", "wasn't very good" etc., and it is not affected by cases where negation flips the sentiment of the text."

Also, we have added the following in the limitation section and page 33:

"Another limitation of our study is that "sarcasm" is challenging to detect in online social media [125]."

Finally, we have added the POS tagging task as future work (please see page 32):

"Fourthly, researchers could examine syntactic habits (e.g. number of verbs or nouns used in a sentence) of entrepreneurs through POS tagging and machine learning techniques."

6. I haven't heard about uClassify before, and it sounds great! What happens if uClassify cannot find a good classification? Naïve Bayes is known for wrongly classifying those borderline cases, which are likely to happen with 10 classes. This would violate the independence assumption of Naïve Bayes. You only kept the tweets with probability higher than .9 to circumvent this problem. How many tweets were disregarded because of this rule? When I look at table 1 and 4, I see for the global sample this amounts to about 20%. With so many disregarded tweets, I can't help but wonder how this might affect the results.

Thank you for this excellent comment. As you correctly mentioned, Naïve Bayes algorithm has some limitations. In order to increase the precision of the uClassify tool and the confidence of the results (assigned topics), we kept only the tweets with topic probability higher than 0.9. Furthermore, it is worth noting that uClassify has been trained on 2.8 million documents with data from Twitter, Amazon product reviews and movie reviews.

In order to test how the reduction of tweets may affect our results, we compared the sentiment of entrepreneurs and non-entrepreneurs per topic a) using all tweets and b) using only tweets with

topic probability higher than or equal to 0.9. The results show that for all datasets entrepreneurs express significantly more positive emotions than non-entrepreneurs on most topics. Overall, our results are robust and remain qualitatively similar.

Table A. Two-sample t-test to compare the mean emotional score per topic between entrepreneurs and non-entrepreneurs (London data). Column A contains the t-test using all tweets, while column B contains only tweets with probability >= 0.9.

Topics	A. All tweets		B. Keep tweets with probability >= 0.9	
	**t**	**Sig. (2-tailed)**	**t**	**Sig. (2-tailed)**
Society	-23.323	0.000	-6.111	0.000
Recreation	-16.058	0.000	-2.773	0.006
Health	-27.618	0.000	-3.461	0.001
Business	-11.992	0.000	-2.861	0.004
Home	-17.207	0.000	-6.284	0.000
Science	-14.297	0.000	-2.742	0.006
Computers	-12.395	0.000	-0.889	0.374
Games	-16.043	0.000	-1.272	0.204
Arts	-52.794	0.000	-8.057	0.000
Sports	-27.182	0.000	-3.716	0.000

Table B. Two-sample t-test to compare the mean emotional score per topic between entrepreneurs and non-entrepreneurs (Los Angeles data). Column A contains the t-test using all tweets, while column B contains only tweets with probability >= 0.9.

Topics	A. All tweets		B. Keep tweets with probability >= 0.9	
	**t**	**Sig. (2-tailed)**	**t**	**Sig. (2-tailed)**
Society	-44.064	0.000	-7.984	0.000
Recreation	-40.446	0.000	-6.673	0.000
Health	-55.376	0.000	-11.870	0.000
Business	-31.736	0.000	-5.036	0.000
Home	-38.190	0.000	-13.429	0.000
Science	-16.726	0.000	-3.167	0.002
Computers	-28.603	0.000	3.651	0.000
Games	-29.782	0.000	-2.783	0.005

Arts	-1.1e+02	0.000	-11.768	0.000
Sports	-42.979	0.000	-5.221	0.000

Table C. Two-sample t-test to compare the mean emotional score per topic between entrepreneurs and non-entrepreneurs (Worldwide data). Column A contains the t-test using all tweets, while column B contains only tweets with probability >= 0.9.

Topics	A. All tweets		B. Keep tweets with probability >= 0.9	
	**t**	**Sig. (2-tailed)**	**t**	**Sig. (2-tailed)**
Society	-37.956	0.000	-10.821	0.000
Recreation	-22.238	0.000	-5.201	0.000
Health	-26.211	0.000	-6.860	0.000
Business	-8.341	0.000	-4.077	0.000
Home	-16.151	0.000	-1.605	0.109
Science	-17.675	0.000	-3.895	0.000
Computers	-27.101	0.000	-5.902	0.000
Games	-10.349	0.000	-5.834	0.000
Arts	-51.899	0.000	-3.574	0.000
Sports	-23.4265	0.000	-2.204	0.028

7. How many tweets per person are included? The regression in table 5 is performed at the person level, while the tables and figures before are at the tweet level. Maybe this could be accentuated when describing the data or results. Also, often some people are very active on twitter, leading to many tweets, which may bias your results. Wouldn't it be fair to only include one tweet per person? Now you basically have multi-level data, which you treat as if it has only one level.

Thank you for this helpful comment. We have clarified the following in the paper.

To avoid any bias in our results, all correlations (table 2, 3 and 4) and regression (table 5) tables are aggregated per user (e.g. avg sentiment, avg hashtags). Furthermore, due to the aggregation process per user, we don't limit the number of tweets. Furthermore, Tables 1, 6 and 10 contain general statistics of the total users and tweets that were used for each analysis. We provide all the necessary clarifications in the text.

Please see below and pages 18-20:

“Tables 2, 3 and 4 report the correlations between the variables for the three samples (at the user level of analysis). “

“Figure 1 plots the overall percentage of positive, negative and neutral tweets both for entrepreneurs and non-entrepreneurs for the three different geographical regions examined (at the tweet level of analysis). As we can observe, the Twitter streams of entrepreneurs contain tweets that are substantially more positive than the tweets of non-entrepreneurs across all samples.”

“Figure 2 plots the percentage of positive tweets published per day of the week, for entrepreneurs and non-entrepreneurs (tweet level). As we can observe, for both user categories, the percentage of positive tweets is lower during weekends, while it increases during weekdays, reaching highest values towards the end of the week. In all cases, entrepreneurs are consistently more positive than non-entrepreneurs for each day of the week.”

“Next, we analyze the emotional score of the two groups during extended periods of time (tweet level). Figure 3 plots the emotional score of each calendar day during a period of 2 years; entrepreneurs are generally more positive than non-entrepreneurs throughout the two-year period.”

“The regression results are shown in Table 5 (at the user level of analysis). The dependent variable is the average sentiment of a user’s tweets.”

Also, please see below and page 28:

“In order to analyze the impact of job changes over time we used fixed-effects regressions. We used the unique identifier for each user as the grouping variable. The panel data are unbalanced as each individual does not have the same number of job changes as others. Table 11 presents the fixed-effects regression estimates (per user level).”

8. Although I appreciate study 2, in combining Twitter data with external data on entrepreneurs, I wonder why the analysis was not conducted at personal level. Now you explore the average sentiment of people on Twitter that claim to be working in a certain job. Even more, your argument resonates with the time dimension in the data, i.e. if people made shifts in their career. You claim that you investigate how these shifts are related to sentiment, but I don't see how you integrated information on the person's career path in the analysis. If possible, it would be nice to include a survival analysis to explore the influence of career shift on sentiment at the person level.

Thank you for this very important comment, which enabled us to clarify our study 2 in the revised paper.

Please see below and page 28:

“In order to analyze the impact of job changes in and out of entrepreneurship over time we used fixed-effects regressions. This enables us to examine whether engagement in entrepreneurship is likely to be associated with the expression of more positive emotions. Specifically, based on the job history and tweets’ sentiment of each user, we ran a within-user (fixed-effects) analysis using the unique identifier for each user as the grouping variable. The panel data are unbalanced as each individual does not have the same number of job changes as others. Table 11 presents the fixed-effects regression estimates. The coefficient for

entrepreneurship is positive and significant ($p<0.01$), indicating that engagement in entrepreneurship is associated with more positive emotions expressed on social media.”

Small comments

1. Top page 5, second paragraph, first sentence. I think the sentence is easier to read when adjusted as such: "To begin, we argue that the act of choosing her work aims and conditions can be enjoyable in itself for an entrepreneur."

We have revised the sentence as suggested. Thank you for this. Please see below and page 6:

“To begin, we argue that the act of choosing her work aims and conditions can be enjoyable in itself for an entrepreneur. Entrepreneurs make a much wider range of choices than employees. Whereas an employee may be offered a precise set of tasks, workplace conditions and working hours as an employment package, an entrepreneur chooses them individually with far greater freedom…”

2. Bottom page 8: "A serial entrepreneur may reflect on these unpleasant prior experiences [2], which are less likely to be considered by less experienced entrepreneurs and which impact negatively the serial entrepreneur's emotions." Can you add time that someone has been an entrepreneur to the analysis as control variable?

Thank you for this comment. Unfortunately, due to the static nature of Twitter profile data (e.g. static profile description), we do not have the time related info for Twitter users.

We have also added the following on page 32:

“Similarly, the time that an individual has been an entrepreneur could be examined as a potential moderator, as it may affect the emotions of an entrepreneur [2].”

3. Table 5. Why not include the two dummy variables for serial and social entrepreneurs in one regression? Now you run two regressions to compare three groups, but you can compare both groups to the reference category (traditional) and suffice with one regression.

Thank you for this excellent suggestion. As suggested, we included two dummy variables, one for social entrepreneurs and one for serial entrepreneurs, in the same regression (please see the revised Table 5 in page 21). The results are the same.

**Sample**	**Entrepreneurs only**
**Region**	**Worldwide (5)**
Entrepreneur	
Social Entrepreneur	.008024***

	(.001619)
Serial Entrepreneur	-.004093
	(.002896)
Number of followers (000s)	2.99e-06
	(8.10e-06)
Number of followings (000s)	.000721***
	(.000081)
Total number of tweets (000s)	-.000275***
	(.000035)
Android source	-.025456***
	(.003806)
Retweets	.002393
	(.002831)
Geotagged tweets	-.064748***
	(.006462)
Hashtags	.010647***
	(.001291)
Constant	.144660***
	(.000694)
N	24,573
R-squared	0.0150
F	41.63***

Notes. The dependent variable is average sentiment based on the Twitter user's tweets. Standard errors are in parentheses.
$^{*}p < .05$; $^{**}p < .01$; $^{***}p < .001$.

Thank you so much for your helpful comments and for sharing your expertise!

Reviewer #3:

Dear authors, your study presents a state-of-the art empirical analysis on the sentiment of entrepreneurs in Twitter messages. I have no issues with the statistical analysis and the results make sense to me. The main novelty is distinguishing between social, serial and for-profit-entrepreneurs as well as the analysis of how job changes by entrepreneurs impact emotions on Twitter.

Thank you very much for your helpful and constructive comments! We are grateful for your comment that our "your study presents a state-of-the art empirical analysis on the sentiment of entrepreneurs in Twitter messages". Your comments have significantly improved the quality of our paper.
Please find below your comments in bold and our responses in plain text.

My main problem is the treatment of prior literature. You cite a lot of general literature about the emotions of entrepreneurs but you largely ignore the more specific (and also well-published) literature on the Twitter sentiments of entrepreneurs.

Here are a couple of papers that are very similar to your paper analyzing emotions of entrepreneurs using Twitter data and comparing them to non-entrepreneurs as well as distinguishing between failed and non-failed entrepreneurs. Please make your positioning and your treatment of prior literature more accurate when you are invited to conduct a revision.

Obschonka, M., Fisch, C., & Boyd, R. (2017). Using digital footprints in entrepreneurship research: A Twitter-based personality analysis of superstar entrepreneurs and managers. Journal of Business Venturing Insights, 8, 13-23.

Obschonka, M., & Fisch, C. (2018). Entrepreneurial personalities in political leadership. Small Business Economics, 50(4), 851-869.

Fisch, C., & Block, J. H. (2020). How does entrepreneurial failure change an entrepreneur's digital identity? Evidence from Twitter data. Journal of Business Venturing,

Thank you for this very helpful comment and for bringing to our attention these excellent papers. We have cited them in the paper. Please see the revised introduction (pages 2-3) and discussion sections (pages 30-31).

For example, on page 31 we mention:

"Our study adds to recent work examining the digital footprints of entrepreneurs. For example, research has studied whether and how entrepreneurs' digital identities change in response to entrepreneurial failure by examining 760 entrepreneurs who experienced failure and their tweets

before, during, and after business failure [26]. The results indicate that the financial, social, and psychological consequences of failure are reflected in entrepreneurs' tweets [26]. Obschonka et al. (2017) [24] compared the digital footprints of 57 superstar entrepreneurs and 49 superstar managers using text analysis based on the individuals' Twitter messages (called tweets) and found significant differences between the personalities of superstar entrepreneurs and managers."

Thank you so much for your helpful comments and for sharing your expertise!

---

6. PLOS authors have the option to publish the peer review history of their article (what does this mean?). If published, this will include your full peer review and any attached files.

If you choose "no", your identity will remain anonymous but your review may still be made public.

Do you want your identity to be public for this peer review? For information about this choice, including consent withdrawal, please see our Privacy Policy.
Reviewer #1: **Yes:** Theresa Treffers
Reviewer #2: **Yes:** Meike Morren
Reviewer #3: No

[NOTE: If reviewer comments were submitted as an attachment file, they will be attached to this email and accessible via the submission site. Please log into your account, locate the manuscript record, and check for the action link "View Attachments". If this link does not appear, there are no attachment files.]

While revising your submission, please upload your figure files to the Preflight Analysis and Conversion Engine (PACE) digital diagnostic tool, https://pacev2.apexcovantage.com/. PACE helps ensure that figures meet PLOS requirements. To use PACE, you must first register as a user. Registration is free. Then, login and navigate to the UPLOAD tab, where you will find detailed instructions on how to use the tool. If you encounter any issues or have any questions when using PACE, please email PLOS at figures@plos.org. Please note that Supporting Information files do not need this step.
