# Supplementary material for: Exploring the sentiment of entrepreneurs on Twitter
Source: PLoS One. 2021 Jul 30;16(7):e0254337. doi: 10.1371/journal.pone.0254337 (PMC8323876; doi:10.1371/journal.pone.0254337)
Supplement: Attachment — Submitted filename: Plos One - Response to Reviewers 10 June.doc [file pone.0254337.s002.doc]

**PONE-D-20-15982R1**

**Exploring the Sentiment of Entrepreneurs on Twitter**

Dear Professor Koellinger,

Thank you so much for requesting a minor revision of our paper, manuscript number PONE-D-20-15982R1, submitted for publication consideration in PLOS One. We are most grateful for the helpful comments that you and the reviewers have provided us in the revision process. Please find the response letter and revised paper attached.

Kind regards,

The Authors

**Thank you for submitting your manuscript to PLOS ONE. After careful consideration, we feel that it has merit but does not fully meet PLOS ONE's publication criteria yet. Therefore, we invite you to submit a revised version of the manuscript that addresses the points raised during the review process.**

**Only a minor revision is required at this point.**

**We look forward to receiving your revised manuscript.**

**Kind regards,**

**Philipp D. Koellinger, Ph.D.**

**Academic Editor**

**PLOS ONE**

Reviewers' comments:

Reviewer's Responses to Questions

**Comments to the Author**

1. If the authors have adequately addressed your comments raised in a previous round of review and you feel that this manuscript is now acceptable for publication, you may indicate that here to bypass the "Comments to the Author" section, enter your conflict of interest statement in the "Confidential to Editor" section, and submit your "Accept" recommendation.

Reviewer #1: All comments have been addressed

Reviewer #2: (No Response)

Reviewer #3: All comments have been addressed

2. Is the manuscript technically sound, and do the data support the conclusions?

The manuscript must describe a technically sound piece of scientific research with data that supports the conclusions. Experiments must have been conducted rigorously, with appropriate controls, replication, and sample sizes. The conclusions must be drawn appropriately based on the data presented.

Reviewer #1: Yes

Reviewer #2: Yes

Reviewer #3: Yes

3. Has the statistical analysis been performed appropriately and rigorously?

Reviewer #1: Yes

Reviewer #2: Yes

Reviewer #3: Yes

4. Have the authors made all data underlying the findings in their manuscript fully available?

The [PLOS Data policy](http://www.plosone.org/static/policies.action#sharing) requires authors to make all data underlying the findings described in their manuscript fully available without restriction, with rare exception (please refer to the Data Availability Statement in the manuscript PDF file). The data should be provided as part of the manuscript or its supporting information, or deposited to a public repository. For example, in addition to summary statistics, the data points behind means, medians and variance measures should be available. If there are restrictions on publicly sharing data—e.g. participant privacy or use of data from a third party—those must be specified.

Reviewer #1: No

Reviewer #2: No

Reviewer #3: Yes

5. Is the manuscript presented in an intelligible fashion and written in standard English?

PLOS ONE does not copyedit accepted manuscripts, so the language in submitted articles must be clear, correct, and unambiguous. Any typographical or grammatical errors should be corrected at revision, so please note any specific errors here.

Reviewer #1: Yes

Reviewer #2: Yes

Reviewer #3: Yes

**6. Review Comments to the Author**

**Reviewer #1: Thank you very much for revising the paper! It reads very well and many constructs have become clearer. I still a few comments to consider:**

**1. From your introduction, it's still not clear to me why we need your study. You state your contributions, but why are they important? For example, why is it important to examine emotion expression instead of experienced emotions? Please elaborate further on your contributions and how and why they are relevant for the field.**

Thank you for this comment. In response to it we added an explanation for the relevance of each contribution: (please see pages 5-6)

“Our work complements earlier studies using Twitter data in managerial or entrepreneurial studies [17,32–34] and makes a number of contributions: Our first contribution is to examine whether there are differences between categories of entrepreneurs (traditional, social and serial entrepreneurs) in their emotional expression on social media platforms like Twitter. Most of the previous research focused on entrepreneurs' emotions but not on the emotional expression of entrepreneurs in social media platforms. It is valuable to understand this expression because it can influence recipients – both by communicating information about the entrepreneur’s views [13] and by aligning the recipient’s and the entrepreneur’s emotional states [14]. In addition, our study expands previous work that considers how emotion influences social entrepreneurship intention and participation [35–41] and shows that social entrepreneurship can lead the entrepreneur to express more positive emotions than other forms of entrepreneurship on social media platforms. We also contribute to the limited literature on emotions in serial entrepreneurship [42,43] by showing that serial entrepreneurship can lead to the expression of less positive emotions than other forms of entrepreneurship. Finally, the study of social media content with big data techniques allows us to conduct analyses at a much larger scale.

Our second contribution is to compare differences in emotions expressed on Twitter between entrepreneurs and non-entrepreneurs in relation to different topics. There is currently limited work examining the effect of entrepreneurship on emotions relating to topics unconnected with entrepreneurship. It is worth examining this link because emotions can affect an entrepreneur’s cognition and judgements [2], which can lead them to act rashly in areas in which they have no expertise. We build on prior literature that has considered emotions specific to certain topics, such as Cardon et al.’s [22] and Gielnik et al.’s [44] examination of emotions towards entrepreneurial identity and activity. By employing 10 different topics, namely "Society", "Recreation", "Health", "Business", "Home", "Science", "Computers", "Games", "Arts", "Sports, we show that entrepreneurs express significantly more positive emotions than non-entrepreneurs in all topics.

Our third contribution is to examine how transition from entrepreneurship to non-entrepreneurship and vice versa can affect the emotions of an individual on social media platforms responding to recent calls [45,46]; we show that engagement in entrepreneurship is associated with a higher emotional positivity. These findings further support the argument that entrepreneurship is associated with more positive emotional expressions on social media platforms, and demonstrate that, controlling for the characteristics of the entrepreneur, entrepreneurial actions are associated with increased positivity. Finally, our study contributes to recent research that has examined the digital footprints of entrepreneurs using computerized text analyses [47][48][49].”

**2. I find your RQs 2 and 3 a bit unspecific and would suggest that you split them into comparisons between the different groups of entrepreneurs, e.g. social entrepreneurs vs serial entrepreneurs. Also, are there serial social entrepreneurs? Or serial entrepreneurs that have started social AND profit-oriented ventures?**

Thank you for this comment. In response to it, we added the following on page 24:

“Also, we compared social versus serial entrepreneurs. We excluded 27 users who were both social and serial entrepreneurs. The results show that social entrepreneurs are significantly more likely to exhibit positive emotions than serial entrepreneurs (p<0.001).”

| **Table - Social vs Serial Entrepreneurs (Social=1, Serial=0)** | |
| --- | --- |
| **Sample** | **Entrepreneurs only** |
| **Region** | **Worldwide** |
| Social Entrepreneur | .011134*** |
|  | (.003399) |
| Number of followers | -.000019 |
| (000s) | (.000046) |
| Number of followings | .000202 |
| (000s) | (.000189) |
| Total number of tweets | .000145 |
| (000s) | (.000091) |
| Android source | -.018820 |
|  | (.013518) |
| Retweets | .008084 |
|  | (.010121) |
| Geotagged tweets | -.062038** |
|  | (.021917) |
| Hashtags | .006436 |
|  | (.003706) |
| Constant | .139168*** |
|  | (.003432) |
|  |  |
| N | 2,074 |
| R-squared | .0128 |
| F | 4.360^***^ |
| Notes. Standard errors in parentheses.  ^*^p < .05; ^**^ p < .01; ^***^ p < .001. | |

**3. I'd love to see a (few) paragraph about the practical implications of our studies. What can you recommend to entrepreneurs, social media platforms, policy makers?**

Thank you for your comment. We have revised this section as follows: (please see page 31)

“Our work also allows us to draw a number of practical implications. One is that entrepreneurship can improve a person’s emotional state, and their emotions directed towards people and events in general. A similar implication is drawn by Kato and Wiklund [117]. Such positive emotions can bring various favourable health outcomes [118], although the benefits of entrepreneurship are tempered by the health and personal consequences of entrepreneurial failure [76,119]. The emotional benefits are weaker for serial entrepreneurs, in part because of their repeated exposure to possible or actual failure. However, the emotional advantages of social entrepreneurship are particularly strong, pointing to both social and personal gains from participation. Overall, there may be additional reasons for governments to encourage engagement in entrepreneurial activities beyond generating employment and economic growth.

Although positive emotions can bring benefits [118], they can also be disadvantageous in a number of ways [120,121]. For example, positive emotions can trigger impulsivity or excessive risk-taking [122]. As entrepreneurs experience unusually positive emotions about other subjects, their emotions may unfavourably impact other aspects of their life, including health, finances, and relationships. We advise entrepreneurs to be aware of this possibility, and adapt a circumspect approach to their own evaluation of matters away from entrepreneurship.

Further, an entrepreneur’s positive emotions in social media may be viewed by their readers and viewers as evidence that the entrepreneur’s organisation is flourishing [13]. They may then invest in or support the organisation, regardless of its economic and operational fundamentals, potentially leading to inefficient allocation of resources as well as transfer of wealth from those investors to the entrepreneur or better-informed economic agents. Investors acting on incomplete or misperceived information has always been an issue, but with fast and direct communication by entrepreneurs through social media, and the resulting sidelining of traditional information intermediaries, the problem is accentuated. It’s not an easy problem to solve; governments and social media companies struggle to control even egregious examples of misinformation, and it seems to be much harder to make people aware of their possibly incorrect inferences based on their possibly unconscious perceptions of an entrepreneur’s emotional state.”

**I really like how you developed your paper further and wish you much success with it!**

Thank you so much for your helpful comments throughout the revision process!

**Reviewer #2: General remark: Although in many parts of the paper, the authors have toned down their claim to measure emotions, here and there this is still mentioned as such. Please read carefully the paper, and replace emotions by expressed emotions on platforms.**

Thank you very much for your helpful comments. They have been very useful in improving the quality of our paper.

In response to your comment, we have replaced emotions by expressed emotions throughout the paper.

**2. The part where you explain why social entrepreneurs express more positive emotions on social media (pages 8-10) is important, but instead of supporting your hypotheses, I would rather use this argumentation as part of the discussion where you suggest explanations (i.e. gratitude among social entrepreneurs) for the effect that you found. In the introduction you can only mention that you expect social entrepreneurs to express more positive emotions. You can mention compassion and motivations but more detailed explanations of this effect (such as gratitude) probably belong better in the discussion. The text I am referring to is:**

**As both traditional entrepreneurs and social entrepreneurs have substantial freedom to set their own goals and working conditions, the arguments that we presented on why traditional entrepreneurs show more positive emotions continue to apply to social entrepreneurs. However, there are additional reasons why social entrepreneurs' emotions may be even more positive than those of traditional entrepreneurs. We propose that social entrepreneurship may lead to altruistic enjoyment of other people's improved circumstances, and so raises the social entrepreneur's emotions. We then argue that social entrepreneurs may receive gratitude from others as they are willing to take on the risk and effort to create positive changes in society through their initiatives, and we see how social entrepreneurship may raise social esteem and enable people to meet social norms, leading to more positive emotion.**

Thank you for this comment. As per your suggestion, we have revised the theoretical development as follows. (please see page 8)

“Our next research question looks at social entrepreneurs, people who apply business practices to address social problems or create social value (there are numerous variations on this definition [62–65]). As both traditional entrepreneurs and social entrepreneurs have substantial freedom to set their own goals and working conditions, the arguments that we presented on why traditional entrepreneurs show more positive emotions continue to apply to social entrepreneurs. Beyond those arguments, we can also note that social entrepreneurs may receive gratitude from others for their activities and gain social esteem. However, it isn’t clear beforehand that these factors would affect social entrepreneurs more than traditional entrepreneurs. Accordingly, we frame our research question without rigid prior theorizing, and later revisit our theory in the light of the observed evidence.”

And we expanded the discussion as follows: (please see pages 29-30)

“We did not previously theorize why social entrepreneurs would exhibit more positive emotions than other entrepreneurs. With the added confidence derived from observing this relation, we can tentatively propose some plausible explanations of how it operates. One explanation could be that social entrepreneurs experience altruistic enjoyment from improvements in other people’s situations [66], leading to feelings of pleasure [67] which are expressed in their social media. Traditional entrepreneurs may also experience these emotions, but they are perhaps weaker than for social entrepreneurs. Another explanation could be that social entrepreneurs demonstrate that they are meeting their social duty or fulfilling norms of behaviour [68,69], and the demonstration can bring increased public esteem, which is weaker for traditional entrepreneurs, and which is reflected in their social media. A further explanation could be that emotions are more important to the decision-making of potential investors in social enterprises, so social entrepreneurs try harder to influence the investors through their own displays of positive emotions. As these explanations are abductively derived, there is no certainty that they are accurate descriptions of the relation’s operation, and further work could help to determine their veracity.”

**4. The difference in expressing emotions between social and the other entrepreneurs seems to resonate with common sense (i.e. doing good rewards in good feelings) but I still struggle with the other entrepreneurs. Why do they score lower on expressing these emotions? Can you explain that? For instance, the following arguments could also apply to non-social entrepreneurs:**

**A social entrepreneur works to create social value in some way and can be considered to forego personal income or leisure in order to devote their time to the activity. [...] Many people will try to demonstrate that they are meeting their social duty or fulfilling norms of behaviour [67, 68], and the demonstration can bring increased public esteem or protect the social entrepreneur from sanction for neglecting their societal duties.**

**I can imagine that the other entrepreneurs feel the same way about showing off profit or other entrepreneurial success. All in all, I agree with your argumentation, but I would like to challenge you to come up with an explanation for why the other entrepreneurs express fewer positive emotions. As R1 suggests, this might also be due to social desirability. If social entrepreneurs score higher on social desirability, then this could also be an alternative explanation. I think a more critical note to the explanation of the effect that is found should be mentioned in the discussion.**

Thanks for this helpful comment. In the theory section, we have removed the inductive explanation for the difference in expressed emotion between social and other entrepreneurs. Within the replacement paragraph, we add a caution that

“….we can also note that social entrepreneurs may receive gratitude from others for their activities and gain social esteem. However, it isn’t clear beforehand that these factors would affect social entrepreneurs more than traditional entrepreneurs. Accordingly, we frame our research question without rigid prior theorizing, and later revisit our theory in the light of the observed evidence.”

In the conclusion we separate the two previously proposed explanations in the inductive analysis, and propose them as distinct abductively derived explanations, with a caution that they are uncertain and that further work is required: (please see pages 29-30)

“With the added confidence derived from observing this relation, we can tentatively propose some plausible explanations of how it operates. One explanation could be that social entrepreneurs experience altruistic enjoyment from improvements in other people’s situations [66], leading to feelings of pleasure [67] which are expressed in their social media. Traditional entrepreneurs may also experience these emotions, but they are perhaps weaker than for social entrepreneurs. Another explanation could be that social entrepreneurs demonstrate that they are meeting their social duty or fulfilling norms of behaviour [68,69], and the demonstration can bring increased public esteem, which is weaker for traditional entrepreneurs, and which is reflected in their social media. … As these explanations are abductively derived, there is no certainty that they are accurate descriptions of the relation’s operation, and further work could help to determine their veracity.”

We add an additional potential explanation for the difference, subject to the same caution: (please see page 30)

“A further explanation could be that emotions are more important to the decision-making of potential investors in social enterprises, so social entrepreneurs try harder to influence the investors through their own displays of positive emotions.”

**6. I appreciate the additional analysis; it is good to know that your results are robust. I do observe that the t values are much higher for all tweets, than only those who have a probability by uClassify of .9 or higher. Could this mean that uClassify has issues with classifying tweets that are very strong in expressing emotions between entrepreneurs and non-entrepreneurs? Why is that? Could you add a remark in the conclusions?**

Thank you very much for your insightful comment. Please see below and page 21:

We also compared the sentiment of entrepreneurs versus non-entrepreneurs per topic using all tweets. Our results remained robust and qualitatively similar. However, we noticed that tweets with topic probability ≥ 0.9 have a lower sentiment than tweets with topic probability < 0.9. To investigate this, we compared the grammatical structure of the tweets. Specifically, we first identified the grammatical terms in each tweet (e.g. number of verbs, nouns, etc.) by using NLTK POS tagger (Python library). Then, we compared these grammatical terms before and after filtering the tweets that have topic probability ≥ 0.9. The results show that tweets with topic probability ≥ 0.9 have more nouns and fewer verbs, adjectives, and adverbs than tweets with topic probability < 0.9. This is expected for topic classification as more nouns improve the topics’ semantic coherence [103]. On the other hand, sentiment analysis tools (e.g. VADER) focus more on verbs, adjectives and adverbs [84][104][105] as nouns are usually treated as semantically empty (i.e. neutral polarity) [106][107].”

| **Table - Comparing grammatical terms before and after topic filtering** | | | | |
| --- | --- | --- | --- | --- |
|  | **Tweets with topic probability < 0.9** | **Tweets with topic probability >= 0.9** | **t** | **Sig. (2-tailed)** |
|  | **Mean** | **Mean** |  |  |
| Sentiment | .3363034 | .2476562 | 73.1924 | 0.0000 |
| #Adjectives | .9097546 | .7833629 | 73.7636 | 0.0000 |
| #Adverbs | .6714403 | .4339024 | 149.0139 | 0.0000 |
| #Nouns | 4.708777 | 5.885706 | -2.7e+02 | 0.0000 |
| #Verbs | 2.172483 | 1.68656 | 170.3499 | 0.0000 |

**As for the other comments, I feel like you addressed them satisfactorily, thank you for your efforts to improve the paper!**

Thank you so much for your helpful comments! They have significantly improved the quality of our paper!

**Reviewer #3: The authors have done a good job. Well done. I am happy to recommend this revised version to be published.**

Thank you so much for your helpful comments! They have significantly improved the quality of our paper!
